# Dynamic gestational week prediction model for pre-eclampsia based on ID3 algorithm

**DOI:** 10.3389/fphys.2022.1035726

**Published:** 2022-10-26

**Authors:** Ziwei Li, Qi Xu, Ge Sun, Runqing Jia, Lin Yang, Guoli Liu, Dongmei Hao, Song Zhang, Yimin Yang, Xuwen Li, Xinyu Zhang, Cuiting Lian

**Affiliations:** ^1^ Faculty of Environment and Life Sciences, Beijing University of Technology, Beijing, China; ^2^ Intelligent Physiological Measurement and Clinical Translation, Beijing International Base for Scientific and Technological Cooperation, Beijing, China; ^3^ Department of Obstetrics, Peking University People’s Hospital, Beijing, China

**Keywords:** hypertensive disorders of pregnancy, pre-eclampsia, decision tree, prediction model, dynamic

## Abstract

Pre-eclampsia (PE) is a type of hypertensive disorder during pregnancy, which is a serious threat to the life of mother and fetus. It is a placenta-derived disease that results in placental damage and necrosis due to systemic small vessel spasms that cause pathological changes such as ischemia and hypoxia and oxidative stress, which leads to fetal and maternal damage. In this study, four types of risk factors, namely, clinical epidemiology, hemodynamics, basic biochemistry, and biomarkers, were used for the initial selection of model parameters related to PE, and factors that were easily available and clinically recognized as being associated with a higher risk of PE were selected based on hospital medical record data. The model parameters were then further analyzed and screened in two subgroups: early-onset pre-eclampsia (EOPE) and late-onset pre-eclampsia (LOPE). Dynamic gestational week prediction model for PE using decision tree ID3 algorithm in machine learning. Performance of the model was: macro average (precision = 76%, recall = 73%, F1-score = 75%), weighted average (precision = 88%, recall = 89%, F1-score = 89%) and overall accuracy is 86%. In this study, the addition of the dynamic timeline parameter “gestational week” made the model more convenient for clinical application and achieved effective PE subgroup prediction.

## 1 Introduction

Hypertensive disorders in pregnancy (HDP) are conditions in which pregnancy and hypertension coexist, with a prevalence of approximately 5%–12% (Mahendra et al., 2021). The pathogenesis of HDP is complex and multifactorial, and although some research has been done, its etiology is still unclear and no effective predictive method has been established. HDP is a multi-causal disease whose pathogenesis is related to impaired placental angiogenesis, placental oxidative stress and abnormal maternal immune response. It is not only hypertension and proteinuria, but especially with the involvement of the heart, lungs, liver and kidneys, the blood, digestive and nervous systems, but also the placenta and the fetus. The disease includes five subtypes: hypertension in pregnancy, PE, eclampsia, chronic hypertension complicated by pre-eclampsia and chronic hypertension in pregnancy (Duhig et al., 2019). PE is one of the more severe of the above sub-types in its pathogenesis. Patients with suspected preeclampsia are diagnosed when any of the following points are met: new onset of hypertension or exacerbation of preexisting hypertension, positive urine test for urine protein, epigastric or right upper abdominal pain, headache with visual disturbance, fetal growth restriction or abnormal maternal blood tests (e.g., thrombocytopenia or liver and kidney dysfunction) (Li et al., 2021). The clinical management of preeclampsia is a complex task for the following reasons: (A) Complex pathogenic background: preeclampsia is a multifactorial-multi-mechanism-multi-pathway pathogenic syndrome. (B) Complex symptom presentation: The degree and presentation of hypertensive symptoms in preeclamptic patients are complex, and the first symptoms are diverse. In traditional medical diagnosis, physicians can only rely on the information of the target patient as well as their own experience and knowledge base to make judgments, which has some limitations. The machine learning approach, however, can better assist in diagnosis. Termination of pregnancy before 34 weeks of gestation due to pre-eclampsia is de-fined as EOPE, and termination at ≥34 weeks of gestation is defined as LOPE (Raymond and Peterson, 2011). Risk factors associated with PE can be divided into various aspects such as clinical epidemiology, hemodynamics, underlying biochemical factors, and biomarkers in pregnant women. If the high-risk risk factors in the development of PE are clarified and a com-prehensive multifactorial dynamic study is performed, the impact and significance of preeclampsia prediction and prevention are very important.

Tan et al. (2020) established a prediction model for severe maternal outcomes in pregnant women with PE by using a multivariable logistic regression model. The model has a good predictive ability by internal validation. Further external validation is required to clarify the clinical applicability of this model. Beth et al. (2014) developed the miniPIERS risk prediction model to provide a simple, evidence-based tool to identify pregnant women in LMICs at increased risk of death or major hypertensive-related complications. The miniPIERS model shows reasonable ability to identify women at increased risk of adverse maternal outcomes associated with the HDP. Saleh et al. (2021) propose a simple clinical prediction model with good discriminative performance to predict the risk of a composite outcome of PE-related maternal and fetal complications within 7, 14, and 30 days of testing in women with suspected or confirmed PE. The clinical pre-diction models with good identification performance can be used to predict PE-related complications. Ziad et al. (2020) using births from 2011 to 2012, multivariable logistic regression incorporated established maternal risk factors to develop and internally vali-date the WS (Western Sydney) model. The WS model was then externally validated using births from 2013 to 2014, assessing its discrimination and calibration. The model achieved modest performance for prediction of PE in nulliparous women but did not outperform the NICE approach.

Placental growth factor (PlGF), a member of the vascular endothelial growth fac-tor family, is a pro-angiogenic factor serum marker with important functions in regulating placental trophoblast and endothelial cell function (Duhig et al., 2020). PlGF levels are usually measured at the first antenatal visit, 11–13 weeks of gestation, 19–24 weeks of gestation, and 30–34 weeks of gestation as a way to assess the risk of developing preeclampsia. However, the pathogenesis of preeclampsia has not been elucidated, and there is a lack of effective clinical means to prevent it. Its multifactorial predisposition, multiple pathways of pathogenesis and individual differences all determine that a single index is not a good predictor of preeclampsia. Researchers have also combined maternal characteristics and the biomarker PlGF to make relevant predictions. Knudsen et al. (2012) demonstrated the potential of the biomarker PlGF as an aid in the diagnosis of PE: the highest clinical sensitivity was calculated using a threshold value based on the fifth percentile of PlGF concentrations in reference pregnancies within a defined gestational week, and the single biomarker PlGF had the same diagnostic performance compared to the ratio of the two biomarkers, simplifying the test results and reducing costs, with some economic benefits. Black et al. (2020) used the Fetal Medicine Foundation algorithm to combine Maternal own condition, mean arterial pressure, mean uterine artery pulsatility index, and median multiples of PlGF parameters for combined screening of mid-pregnancy PE. Stepan et al. (2020) combined information from ultrasound, mean arterial pressure, clinical features and PlGF to improve the prediction of PE in early pregnancy. Poon et al. (2009) used a logistic regression analysis algorithm combining mean arterial pressure, uterine artery pulsatility index, PAPP-A and PlGF for the prediction of preeclampsia and its subtypes. Mendoza et al. (2021) Combined screening for PE and its subtypes in early pregnancy with physical indicators and biomarkers. Sufriyana et al. (2020) predicted PE by maternal characteristics, uterine motility Doppler measurements, sFlt-1 and PlGF in mid and late pregnancy. sFlt-1 and PlGF in mid- and late-trimester, using machine learning-related algorithms to predict PE.

The aim of this study was to develop and validate a model for predicting the risk of PE for uncomplicated pregnancies. The model can be used at prenatal visits at different gestational weeks to predict whether a pregnant woman is likely to have PE and if so whether she has EOPE or LOPE.

## 2 Materials and methods

The study data were obtained from clinical epidemiological data, hemodynamic data, data on underlying biochemical data, and biomarker data. Radial artery and fingertip volumetric pulse waveform information collected from 2015 to 2016 at Beijing Haidian District Maternal and Child Health Hospital and from 2006 to 2008 at Beijing Maternity Hospital for detecting gestational weeks of 10–40 weeks. The clinical epidemiological data, hemodynamic data, data on underlying biochemical data, and biomarker data, and PlGF parameter information were collected from July 2015 to 2017 at Peking University People’s Hospital for the detection of gestational weeks 10–40 weeks. And PlGF testing was mainly focused on about 15–26 weeks. The study subjects were included in the following conditions: pregnant women were not on long-term oral medication; The fetus was free of malformations.

The study population was 80 pregnant women with EOPE (96 tests), 219 pregnant women with LOPE (371 tests) and 633 pregnant women without HDP (1,351 tests). Pregnant women with EOPE were included in the EOPE group, those with LOPE in the LOPE group, and those without HDP in the control group.

### 2.1 Model parameter filtering

Risk factors for PE mainly include clinical epidemiological factors, hemodynamic factors, basic biochemical factors and biomarker factors. In order to analyze the correlation dynamics of each model parameter before the construction of the dynamic gestation prediction model of PE, and according to whether the factors themselves change with the gestational age, the risk factors initially screened out are divided into static parameters that do not change with the gestational age and dynamic parameters that change with the gestational age. This is shown in Table 1.

**TABLE 1:** Classification of PE risk factors.

Category		Factors
Static parameters	Qualitative factors	First birth, multiple births, spontaneous miscarriage history, history of hypertension in pregnancy, history of diabetes mellitus, family history of hypertension, family history of diabetes mellitus, gestational diabetes mellitus, pregestational diabetes mellitus, pregnancy with immune system diseases, pregnancy with hematologic diseases, pregnancy with thyroid diseases
	Quantitative factors	Height, age, preconception body mass index
Dynamic parameters	Epidemiological factors	body mass index during pregnancy
	Hemodynamic factors	Systolic blood pressure (SBP), diastolic blood pressure (DBP), pulse pressure (PP), mean arterial pressure (MAP), pulse waveform area parameters(K), cardiac output (CO), cardiac index (CI), total peripheral resistance (TPR)
	Basic biochemical factors	Hematocrit (HCT), mean platelet volume (MPV), platelet count (PLT), alanine aminotransferase (ALT), aspartate aminotransferase (AST), creatinine (CRE), uric acid (UA)
	Biomarker factors	PlGF

The characteristic parameters of pulse wave were obtained by detecting the pulse wave of radial artery. Radial artery pulse wave detection at the Beijing Obstetrics and Gynecology Hospital was obtained by MP HDP detection instrument developed by Beijing Yes Medical Devices Co., Ltd. The eight-channel PowerLab data acquisition system, LabChart 8 software and strain gauge pressure sensor were used to collect radial artery pulse wave at the Beijing Haidian Maternal and Child Health Hospital. Biochemical parameters were obtained by blood routine examination and biochemical examination. SPSS 23.0 software was used for statistical basic analysis and decision tree was used to construct the predictive model of PE in Jupyter Notebook.

#### 2.1.1 Static parameter filtering

For the screening of static parameters that do not change with the gestational age, the basic information statistics of qualitative and quantitative parameters are used. In order to describe the difference between the parameters in the EOPE group and the LOPE group and the same control group. A chi-square test was performed on 12 qualitative factors. Odds ratio (OR) > 1, indicating that the risk of this factor associated with PE was high, and *p* < 0.05 was statistically significant. While the independent sample *t* test for three quantitative factors was expressed as mean ± standard deviation, and *p* < 0.05 was statistically significant. The specific analysis of static parameters of pregnant women in the EOPE group, LOPE group and the same control group that do not change with gestational age is shown in Tables 2, 3.

**TABLE 2:** Analysis of factors that do not change with gestational age in EOPE subgroup and control.

Parameter	EOPE subgroup	Control group	OR
Number	80	633	—
First birth	56 (70.0%)	515 (81.4%)	0.535
Multiple births	7 (8.8%)**	4 (0.6%)	15.079
Spontaneous miscarriage history	39 (48.8%)**	141 (22.3%)	3.319
History of hypertension in pregnancy	2 (2.5%)*	1 (0.2%)	16.205
History of diabetes mellitus	2 (2.5%)	10 (1.6%)	1.597
Family history of hypertension	15 (18.8%)	105 (16.6%)	1.160
Family history of diabetes mellitus	2 (2.5%)	32 (5.1%)	0.482
Gestational diabetes mellitus	2 (2.5%)	37 (5.8%)	0.413
Pregestational diabetes mellitus	0	2 (0.3%)	0.997
Pregnancy with immune system diseases	2 (2.5%)	14 (2.2%)	1.134
Pregnancy with hematologic diseases	2 (2.5%)	20 (3.2%)	0.786
Pregnancy with thyroid diseases	2 (2.5%)	30 (4.7%)	0.515
Age	30.650 ± 4.543	30.220 ± 3.742	—
Height(m)	1.618 ± 0.051	1.624 ± 0.048	—
Preconception body mass index	55.734 ± 8.588**	21.140 ± 3.101	—

Notes: *for *p* < 0.05, ** for *p* < 0.001. *p* < 0.001 has significant difference.

**TABLE 3:** Analysis of factors that do not change with gestational age in LOPE subgroup and control group.

Parameter	LOPE subgroup	Control group	OR
Number	219	633	—
First birth	172 (78.5%)	515 (81.4%)	0.839
Multiple births	12 (5.5%)**	4 (0.6%)	9.116
Spontaneous miscarriage history	90 (41.1%)**	141 (22.3%)	2.434
History of hypertension in pregnancy	6 (2.7%)**	1 (0.2%)	17.803
History of diabetes mellitus	7 (3.2%)	10 (1.6%)	2.057
Family history of hypertension	51 (23.3%)*	105 (16.6%)	1.527
Family history of diabetes mellitus	24 (11.0%)*	32 (5.1%)	2.312
Gestational diabetes mellitus	12 (5.5%)	37 (5.8%)	0.934
Pregestational diabetes mellitus	1 (0.5%)	2 (0.3%)	1.447
Pregnancy with immune system diseases	9 (4.1%)	14 (2.2%)	1.895
Pregnancy with hematologic diseases	2 (0.9%)	20 (3.2%)	0.282
Pregnancy with thyroid disease	8 (3.7%)	30 (4.7%)	0.762
Age	30.350 ± 4.300	30.220 ± 3.742	—
Height(m)	1.619 ± 0.053	1.624 ± 0.048	—
Preconception body mass index	23.239 ± 3.916**	21.140 ± 3.101	—

Notes: *for *p* < 0.05, ** for *p* < 0.001. *p* < 0.001 has significant difference.

The static parameters of 80 cases of EOPE group and 633 control groups that did not change with gestational age were as follows: multiple pregnancies, history of spontaneous abortion, and history of hypertensive disease during pregnancy were qualitative parameters, and the proportion of all in the EOPE subgroup was higher than that of the control group, OR>1 and *p* < 0.05, indicating that these factors were high-risk and statistically significant; preconception body mass index was a quantitative parameter, which was significantly higher than that of the control group in the EOPE subgroup, and *p* < 0.001 was statistically significant, as shown in Table 2. Multiple pregnancies, a history of spontaneous miscarriage, a history of hypertensive disorders during pregnancy, and a history of preconception body mass index as static parameters in the EOPE subgroup are consistent with clinical needs and previous studies.

Static parameter analysis of 219 patients in the LOPE group and 633 control groups that did not change with gestational age: multiple pregnancies, natural abortion history, gestational hypertension disease history, hypertension family history, and diabetes family history, the proportion of the LOPE group was higher than that of the control group. OR >1 and *p* < 0.05 indicated that the risk of factors was high and statistically significant; preconception BMI was a quantitative parameter, which was significantly higher than that of the control group in the LOPE group, and *p* < 0.001 was statistically significant, as shown in Table 3. The inclusion of multiple pregnancies, history of spontaneous abortion, history of hypertensive disease during pregnancy, family history of hypertension, family history of diabetes and preconception body mass index as static parameters of the LOPE subgroup has certain significance from the perspective of clinical and related research (Knudsen et al., 2012; Black et al., 2020; Duhig et al., 2020).

#### 2.1.2 Dynamic parameter filtering

For the screening of dynamic parameters that change with gestational age, the control variable analysis is mainly carried out. This study mainly constructs a dynamic gestational age prediction model, and the selection of dynamic parameters that change with gestational age takes into account the improvement of the dynamic model effect, etc., and the clinical epidemiological factors that change with gestational age mentioned in Table 1 above: gestational body mass index and one of the effective biomarkers (PlGF) These two dynamic parameters, which are relatively small in this study, are directly included in the model, and are also consistent with clinical needs and previous studies (Rantakallio et al., 2021). The purpose of combining hemodynamic factors is that hemodynamic alterations are important factors in the development and progression of preeclampsia in patients with preeclampsia due to various pathophysiological alterations resulting in blood concentration, decreased blood volume, and increased peripheral resistance. Blood pressure is the combined result of the interaction of hemodynamic parameters. SBP and DBP are obtained from clinical history data, and PP indicates that pulse pressure difference is related to both SBP and DBP. MAP is the mean value of arterial blood pressure during a cardiac cycle, CI mainly reflects cardiac function-related conditions, CO is a very important blood flow parameter to assess cardiovascular function, and TPR can measure small vessel spasm.

For a total of 15 parameters of hemodynamic factor(H) and basal biochemical factor(B) in Table 1 that change with gestational age, the control variables were analyzed in two groups, that is, the probability of parameter combination was calculated by logistic regression to control the parameters of other classes within a fixed range, and the two types of parameters were then independently sampled *t*-tested and outliers analyzed at different gestational stages.

Finally, the parameters for inclusion in the prediction model of each dynamic subgroup were finally determined based on the actual needs of clinical and related studies. Finally, the parameters for inclusion in the prediction model of each dynamic subgroup were finally determined based on the actual needs of clinical and related studies. From the perspective of clinical and related research, further group analysis of gestational segments was carried out at 20 weeks and 34 weeks in the first and third trimesters of each group, namely the second-trimester-E group (ST-EG) and the second-trimester-L group (ST-LG), as well as the late-trimester-E group (LT-EG) and the late-trimester-L group (LT-LG) (Meah et al., 2016). If there are multiple tests in the group, the data of the later and earlier detection of the second and late trimesters of pregnancy are taken respectively to focus on the changes in the parameters of the second half of the late trimester and the first half of the late trimester affect whether the final pregnant woman is ill. The sensitivity analysis of the EOEP subgroup and the LOPE subgroup for the H and B parameters of each gestational segment is shown in Tables 4, 5, and the values of each parameter represent the mean of the *t* test of the independent samples.

**TABLE 4:** Sensitivity analysis of H and B parameters in EOPE subgroup and control group.

Parameter	Group	ETG	ST-EG	ST-LG	LT -EG	LT-LG
SBP	EOPE subgroup	114.438	124.286*	120.000*	118.385*	148.963*#
	Control group	115.547	112.313	109.252	108.252^1^	110.013
DBP	EOPE subgroup	75.438	77.857*	77.316*	75.538*	96.074*#
	Control group	73.795	70.270	68.454	68.034	69.479
PP	EOPE subgroup	39.000	46.429	42.684	42.846	52.889*#
	Control group	41.752	42.043	40.797	40.218	40.534
MAP	EOPE subgroup	91.553	95.680*	93.128*	91.603*	117.952*
	Control group	90.336	86.029	83.583	84.195	84.453
K	EOPE subgroup	0.414#	0.387	0.373	0.385*	0.415*#
	Control group	0.401#	0.380	0.375	0.373	0.403#
CO	EOPE subgroup	4.071	5.286	5.181	5.215	5.273*
	Control group	4.320	4.795	4.876	4.969	4.302
CI	EOPE subgroup	2.573	3.088	3.203	3.174	2.960*
	Control group	2.781	3.048	3.006	3.000	2.547
TPR	EOPE subgroup	1.494#	1.121#	1.112	1.410*#	1.105
	Control group	1.331#	1.136#	1.088	1.079	1.267#
HCT	EOPE subgroup	37.321	38.512*	38.329*	35.985	37.654
	Control group	37.591	35.246	35.197	36.129	36.175
MPV	EOPE subgroup	8.902	9.106	9.692	9.678	10.477*
	Control group	8.986	9.277	9.615	9.664	9.203
PLT	EOPE subgroup	228.482	241.275	192.046	180.378	180.923
	Control group	222.409	221.554	205.166	196.200	199.134
ALT	EOPE subgroup	19.669	20.446	21.886	23.333*	23.500
	Control group	23.786	21.423	22.692	21.814	22.765
AST	EOPE subgroup	21.206	21.964	22.694	23.889*	24.000
	Control group	23.710	22.497	23.175	22.735	23.563
CRE	EOPE subgroup	47.761	62.517	62.098	76.275*	56.395
	Control group	52.227	61.248	65.818	49.743	54.863
UA	EOPE subgroup	212.681	231.447	232.684*	335.053*	276.247
	Control group	200.542	240.577	246.082	228.289	263.255

Notes: * indicates *p* < 0.05 between groups, # indicates outside the normal range.

**TABLE 5:** Sensitivity analysis of H and B parameters in LOPE subgroup and control group.

Parameter	Group	ETG	ST-EG	ST-LG	LT-EG	LT-LG
SBP	LOPE subgroup	114.118	120.226*	120.039*	127.897*	136.083*
	Control group	115.575	112.009	109.117	110.067	109.280
DBP	LOPE subgroup	73.647	76.547*	74.471*	80.971*	90.861*#
	Control group	73.856	70.164	68.400	69.537	68.986
PP	LOPE subgroup	40.471	43.679	45.569*	46.926*	45.222*
	Control group	41.719	41.845	40.717	40.530	40.294
MAP	LOPE subgroup	90.119	92.526*	91.526*	98.891*	108.468*
	Control group	90.369	85.847	83.510	84.510	84.928
K	LOPE subgroup	0.407#	0.374	0.379	0.383*	0.387
	Control group	0.401#	0.380	0.375	0.373	0.397
CO	LOPE subgroup	4.313	4.900	5.248	5.424*	5.206*
	Control group	4.322	4.796	4.867	4.968	4.458
CI	LOPE subgroup	2.774	3.183	3.195	3.201*	2.927*
	Control group	2.782	3.055	3.005	3.003	2.627
TPR	LOPE subgroup	1.347#	1.149	1.118	1.152	1.339#
	Control group	1.331#	1.134	1.090	1.080	1.238#
HCT	LOPE subgroup	37.584	36.492*	36.633*	36.976	36.972
	Control group	37.588	35.196	35.173	36.079	36.586
MPV	LOPE subgroup	8.943	9.710*	9.403	9.791	10.297*
	Control group	8.987	9.285	9.600	9.660	9.411
PLT	LOPE subgroup	223.983	201.779*	203.542	193.047	179.028
	Control group	222.049	220.816	205.503	195.800	194.364
ALT	LOPE subgroup	19.394	18.746*	22.673	22.334	23.500
	Control group	23.788	21.506	22.769	21.834	22.888
AST	LOPE subgroup	20.782	19.882*	22.845	23.326	24.000
	Control group	23.708	22.572	23.215	22.754	23.636
CRE	LOPE subgroup	48.396	53.677*	65.454	65.037*	61.924*
	Control group	52.211	61.427	65.848	49.630	55.067
UA	LOPE subgroup	203.576	211.578*	238.838*	304.248*	308.173*
	Control group	200.624	240.662	246.135	228.304	265.950

Notes: * indicates *p* < 0.05 between groups, # indicates outside the normal range.

The results in Tables 4, 5 showed that the H and B parameters of each gestational segment met the conditions of sensitive parameters in the LOPE subgroup, that is, the prediction of disease outcomes was more sensitive, and the platelet count of the EOPE subgroup did not meet the sensitive parameter conditions, possibly because the amount of data in the EOPE subgroup was relatively small and did not reflect significant differences or abnormalities. However, the two are themselves dynamic parameters.

In this study, the H and B parameters are put into the prediction model of each dynamic subgroup. In addition to the model parameters summarized above, the gestational age as a timeline parameter is also directly incorporated into the prediction model to form a dynamic model. Conditions met for sensitive parameters: independent samples *t*-test for parameters, that is, *p* < 0.05 between groups in the disease and control groups or parameters outside the range of normal values.

#### 2.1.3 Final parameters

Through the filtering of dynamic and static parameters, the parameters identified for inclusion in this study are shown in Table 6.

**TABLE 6:** Parameters eventually incorporated into the model.

Category	Factors
Static parameters	Multiple births, spontaneous miscarriage history, history of hypertension in pregnancy, history of diabetes mellitus, family history of hypertension, preconception body mass index
Dynamic parameters	gestational week, body mass index during pregnancy, SBP, DBP, PP, MAP, K, CO, CI, TPR, HCT, MPV, PLT, ALT, AST, CRE, UA, and PlGF

### 2.2 Machine learning model

The study used an algorithm from decision trees called the Iterative Dichotomiser (ID3) algorithm (Quilan, 1986). The algorithm is a classification prediction algorithm proposed by J. Ross Quinlan at the University of Sydney in 1975. The ID3 algorithm calculates the information gain of each label by selecting the attribute with the highest information gain as the classification criterion for each division, and repeats the process until a perfect decision tree can be generated.

Information entropy is a metric to measure the purity of a sample set. Suppose that the proportion of the class k sample in the sample set D is $p_{k}\left(k=1,2,3\ldots \left|y\right|\right)$ , then the entropy of the information D is:

$$Ent\left(D\right)=-\overset{\left|y\right|}{\underset{k=1}{\sum }}p_{k}\log_{2}p_{k}$$

Assuming that the discrete attribute a has v possible values, if a is used to divide the data set D , v branch nodes are generated, wherein the v branch node contains all the samples in D with the value of $a^{v}$ on the property a , denoted as $D^{v}$ . According to Equation, the information entropy of $D^{v}$ is calculated, and the number of samples contained in different branch nodes is taken into account, and the branch nodes are given weights $\frac{\left|D^{v}\right|}{\left|D\right|}$ , that is, the greater the influence of branch nodes with more sample numbers, so the information gain obtained by dividing sample D by the a attribute is:

$$Gain\left(D,a\right)=Ent\left(D\right)-\overset{V}{\underset{v=1}{\sum }}\frac{\left|D^{v}\right|}{\left|D\right|}Ent\left(D^{v}\right)$$

The process of the algorithm is as follows:

1) Classification training starts from the root node, calculates the information gain of all possible features, and selects the feature with the largest information gain as the partition feature of the node;
2) Child nodes are established from different values for the feature;
3) Recursive step 1 to step 2 of the child nodes to construct the decision tree;
4) A final decision tree is obtained until no features can be selected or the categories are identical.

## 3 Results

The dataset is shown in Table 7. Using 70%/30% random training/test data splitting, repeat this process 20 times and achieve average performance. The model should classify and predict EOPE, LOPE and healthy people.

**TABLE 7:** The dataset of the model.

	EOPE	LOPE	Health	Total
Training set	68	255	949	1,272
Test set	28	116	402	546
Total	96	371	1,351	1818

Precision, recall, and F1-score are used as evaluation indicators for this model. For evaluating performance average across categories, there are two conventional methods, namely macro average and weighted average. Macro averaged performance scores are computed by first computing the scores for the per-category contingency tables and then averaging these per-category scores to compute the global means (Yang et al., 1999). When there is a serious class imbalance in the dataset, the weighted average can be adopted. The performance of the model is shown in Table 8, Overall accuracy of the model is 86%.

**TABLE 8:** The performance of the model.

	Precision (%)	Recall (%)	F1-score (%)
macro average	76	73	75
weighted average	88	89	89

## 4 Discussion

This study describes the importance of predicting PE, and analyzes the status of existing relevant studies comparing risk factors and prediction methods for PE and other deficiencies, thus illustrating the need and importance of this study. This study is mainly based on retrospective analysis and screening of factors that are effective for the risk of developing EOPE as well as LOPE by combining four categories of factors: clinical epidemiological factors, hemodynamic factors, basic biochemical factors and biomarkers. Based on the model parameters obtained from the screening of each subgroup, the decision tree (ID3) algorithm was used to develop dynamic gestational week prediction models for two types of subgroups, EOPE and LOPE, respectively. The core idea of the ID3 algorithm is to measure the selection of attributes in terms of information gain and select the attribute with the greatest information gain after splitting for splitting. The algorithm uses a top-down search to traverse the space of possible decisions. In other words, before dividing each non-leaf node of the decision tree, the information gain of each risk factor incorporated into the model is calculated, and then the risk factor with the greatest information gain is selected for division, because the greater the information gain, the more representative the risk factor is, and the stronger the algorithm’s ability to identify early-onset pre-eclampsia. The model structure was optimized and simplified to enhance the clinical applicability of the model in order to achieve detailed and effective prediction using a simpler dynamic periconceptional subgroup model.

There are still many ways to predict PE Carhillon et al. (2005). Showed that measuring umbilical artery flow parameters such as peak systolic velocity/end diastolic (S/D), beat index, and resistance index can predict the occurrence of PE. In urine, there are studies on the use of urine proteomics for the diagnosis and screening of PE (Carty et al., 2011). Proteomic analysis of the cerebrospinal fluid can accurately determine the severity of PE (Norwitz et al., 2011). sFlt-1 is an anti-angiogenic factor serum marker that downregulates and inhibits the bioactivity of PIGF in promoting placental vascular growth. sFlt-1/PlGF ratio is a good predictive value and diagnostic guide for PE when measured jointly by Bian et al. (2019). However, single prediction is one-sided and unstable, Cnossen et al. (2008) performed a separate study of uterine artery Doppler and the results were low for PE-related subtypes The predictive value of PE-related subtypes was low.

In this study, a multifactorial PE subgroup analysis was performed by combining four categories of clinical epidemiological factors, hemodynamic factors, basal biochemical factors and biomarkers, reclassified according to whether they varied with gestational week. Among them, the biomarker PlGF was tested and compared and had a more significant predictive role and value for the EOPE subgroup. The biomarker is an important dynamic parameter, and the current testing gestational weeks of PlGF in this subject are mainly distributed in 15–26 weeks, with less data on testing samples in the rest of the gestational weeks. In order to improve the quality and effectiveness of the full gestational week data model, the clinical data of full gestational week testing of the biomarker PlGF need to be supplemented in the future. The data in this study are based on retrospective analysis and have limitations such as the type of data. To achieve reliable prediction and enhance clinical application, prospective and multicenter studies are needed to demonstrate the clinical utility of predictive parameters.

## 5 Conclusion

In this study, a multifactorial approach was used for the prediction of dynamic PE-related subgroups, and the model was further refined and incorporated the dynamic timeline parameter “gestational week” for overall dynamic gestational week prediction. It is simpler and more convenient for clinical application, and the model parameters and structure are optimized to achieve effective PE subgroup prediction. This study’s model and method for the prediction of PE integrated dynamic gestational week subgroups is of great significance in giving targeted clinical predictions and recommendations for improving maternal and infant conditions.

## Data Availability

The original contributions presented in the study are included in the article/supplementary material, further inquiries can be directed to the corresponding author.

## References

[B1] Beth A. Jennifer A. Ansermino J. M. Hall D. R. Bhutta Z. A. Bhutta S. Z. (2014). A risk prediction model for the assessment and triage of women with hypertensive disorders of pregnancy in low-resourced settings: The miniPIERS (Pre-eclampsia integrated estimate of RiSk) multi-country prospective cohort study. PLoS Med. 11, 10015899–e1001613. 10.1371/journal.pmed.1001589 PMC3897359 24465185

[B2] Bian X. Biswas A. Huang X. Lee K. J. Li T. K. T. Masuyama H. (2019). Short-term prediction of adverse outcomes using the sFlt-1 (soluble fms-like tyrosine kinase 1)/PlGF (placental growth factor) ratio in asian women with suspected preeclampsia. Hypertension 74, 164–172. 10.1161/HYPERTENSIONAHA.119.12760 31188674 PMC6587370

[B3] Black C. Rolnik D. Al-Amin A. Kane S. C. Stolarek C. White A. (2020). Prediction of preterm pre-eclampsia at midpregnancy using a multivariable screening algorithm. Aust. N. Z. J. Obstet. Gynaecol. 60, 675–682. 10.1111/ajo.13113 32124434

[B4] Carhillon L. Ziol M. Challier J. C. Perrot N. Uzan M. Prevot S. (2005). Doppler and immunohistochemical evaluation of decidual spiral arteries in early pregnancy. Gynecol. Obstet. Invest. 59, 24–28. 10.1159/000080671 15627778

[B5] Carty D. Siwy J. Brennand J. Zurbig P. Mullen W. Franke J. (2011). Urinary proteomics for prediction of preeclampsia. Hypertension 57, 561–569. 10.1161/HYPERTENSIONAHA.110.164285 21199994

[B6] Cnossen J. Morris R. Ter G. Mol B. W. J. van der Post J. A. M. Coomarasamy A. (2008). Use of uterine artery Doppler ultrasonography to predict preeclampsia and intrauterine growth restriction: A systematic review and bivariable meta-analysis. Can. Med. Assoc. J. 178, 701–711. 10.1503/cmaj.070430 18332385 PMC2263112

[B7] Duhig K. Webster L. Sharp A. Gill C. Seed P. T. Shennan A. H. (2020). Diagnostic accuracy of repeat placental growth factor measurements in women with suspected preeclampsia: A case series study. Acta Obstet. Gynecol. Scand. 99, 994–1002. 10.1111/aogs.13818 32017014 PMC7496131

[B8] Duhig K. E. Myers J. Seed P. T. Sparkes J. Lowe J. Hunter R. M. (2019). Placental growth factor testing to assess women with suspected pre-eclampsia: A multicentre, pragmatic, stepped-wedge cluster-randomised controlledtrial. Lancet 393, 1807–1818. 10.1016/S0140-6736(18)33212-4 30948284 PMC6497988

[B9] Knudsen U. Kronborg C. von Dadelszen P. Kupfer K. Lee S. W. Vittinghus E. (2012). A single rapid point-of-care placental growth factor determination as an aid in the diagnosis of preeclampsia. Pregnancy Hypertens. 2, 8–15. 10.1016/j.preghy.2011.08.117 26104984

[B10] Li F. Qin J. Zhang S. Chen L. (2021). Prevalence of hypertensive disorders in pregnancy in China: A systematic review and meta-analysis. Pregnancy Hypertens. 24, 13–21. 10.1016/j.preghy.2021.02.001 33626437

[B11] Mahendra V. Clark S. Suresh M. S. (2021). Neuropathophysiology of preeclampsia and eclampsia: A review of cerebral hemodynamic principles in hypertensive disorders of pregnancy. Pregnancy Hypertens. 23, 104–111. 10.1016/j.preghy.2020.10.013 33310389

[B12] Meah L. Cockcroft J. Backx K. Shave R. Stohr E. J. (2016). Cardiac output and related haemodynamics during pregnancy: A series of meta-analyses. Heart 102, 518–526. 10.1136/heartjnl-2015-308476 26794234

[B13] Mendoza M. Garcia-Manau P. Arevalo S. Aviles M. Serrano B. Sanchez-Duran M. A. (2021). Diagnostic accuracy of first-trimester combined screening for early-onset and preterm pre-eclampsia at 8-10 compared with 11-13 weeks' gestation. Ultrasound Obstet. Gynecol. 57, 84–90. 10.1002/uog.22071 32388877 PMC7839448

[B14] Norwitz E. Tsen L. Park J. Fitzpatrick P. A. Dorfman D. M. Saade G. R. (2011). Discriminatory proteomic biomarker analysis identifies free hemoglobin in the cerebrospinal fluid of women with severe preeclampsia. Am. J. Obstet. Gynecol. 193, 957–964. 10.1016/j.ajog.2005.06.055 16157094

[B15] Poon L. Kametas N. Maiz N. Akolekar R. Nicolaides K. H. (2009). First-trimester prediction of hypertensive disorders in pregnancy. Hypertension 53, 812–818. 10.1161/HYPERTENSIONAHA.108.127977 19273739

[B16] Quilan J. (1986). Induction of decision trees. Mach. Learn. 4, 81–106. 10.1007/bf00116251

[B17] Rantakallio J. Nevalainen J. West S. I. Ollila M. M. Puukka K. Bloigu A. H. (2021). Association of self-reported polycystic ovary syndrome, obesity, and weight gain from adolescence to adulthood with hypertensive disorders of pregnancy: A community-based approach. Hypertension 77, 1010–1019. 10.1161/HYPERTENSIONAHA.120.15702 33517680

[B18] Raymond D. Peterson E. (2011). A critical review of early-onset and late-onset preeclampsia. Obstet. Gynecol. Surv. 66, 497–506. 10.1097/OGX.0b013e3182331028 22018452

[B19] Saleh L. Alblas M. M. Nieboer D. Neuman R. I. Vergouwe Y. Brusse I. A. (2021). Prediction of pre-eclampsia-related complications in women with suspected or confirmed pre-eclampsia: Development and internal validation of clinical prediction model. Ultrasound Obstet. Gynecol. 58, 698–704. 10.1002/uog.23142 33030757 PMC8596877

[B20] Stepan H. Hund M. Andraczek T. (2020). Combining biomarkers to predict pregnancy complications and redefine preeclampsia the angiogenic-placental syndrome. Hypertension 75, 918–926. 10.1161/HYPERTENSIONAHA.119.13763 32063058 PMC7098437

[B21] Sufriyana H. Wu Y. Su E. (2020). Prediction of preeclampsia and intrauterine growth restriction: Development of machine learning models on a prospective cohort. JMIR Med. Inf. 8, 15411. 10.2196/15411 PMC7265111 32348266

[B22] Tan J. Yang M. Liao Y. Qi Y. Ren Y. Liu C. (2020). Development and validation of a prediction model on severe maternal, outcomes among pregnant women with pre-eclampsia: A 10-year cohort study. Sci. Rep. 10, 15590. 10.1038/s41598-020-72527-0 32973289 PMC7518280

[B23] Yang Y. Fischer P. Leu S. J. Zhu M. Woods V. L. Jr Chen P. P. (1999). Possible presence of enhancing antibodies in idiopathic thrombocytopenic purpura. Br. J. Haematol. 1, 69–80. 10.1046/j.1365-2141.1999.01144.x 10027714

[B24] Ziad T. Malcolm H. Jenkins G. Mahmoud I. Ray J. G. Askie L. M. (2020). Prediction of pre-eclampsia in nulliparous women using routinely collected maternal characteristics: A model development and validation study. BMC Pregnancy Childbirth 20, 23–14. 10.1186/s12884-019-2712-x 31906891 PMC6945640

